# Supplementary material for: Obesity-Linked PPARγ Ser273 Phosphorylation Promotes Beneficial Effects on the Liver, despite Reduced Insulin Sensitivity in Mice
Source: Biomolecules. 2023 Mar 31;13(4):632. doi: 10.3390/biom13040632 (PMC10135908; doi:10.3390/biom13040632)

Supplementary Material:

Obesity-linked PPARg Ser273 phosphorylation promotes beneficial effects on the liver, despite reduced insulin sensitivity in mice.

Terra, M.F.[1,2], García-Arévalo, M.[1], Avelino, T.M.[1,3], Degaki, K.Y.[1], de Carvalho, M.[1,4], Torres, F.R.[1], Saito, A.[1], Figueira, A.C.M.[1*]

Figure S1. CRISPR/Cas9 strategy for *knockin* (KI) mice lineage development. (A) Strategy for the development of *knockin* mice lineage, with PPARγ gene and its respective exons (blackboxes) highlighting Ser273 (TCA codon) in the fifth exon. In thewild-type (WT) allele, the sgRNA target sequence is shown in blue, Protospacer Adjacent Motif (PAM) in light orange and Ser273 in red. The single-stranded oligodeoxynucleotide (ssODN) donor oligo contains the S273A missense mutation in red, silent mutations in blue to prevent Cas9 cleaving after homologous recombination and a ScaI restriction enzyme site for genotyping. (B) Enzyme restriction assay in which wild-type animals contain only one 627 bpfragment, whereas heterozygous contain both 627 and 427 bp, and homozygous contain 427 and 200 bp bands. (C) Electropherograms of wild-type and homozygous animals, highlightingSer273 and enzyme restriction points.

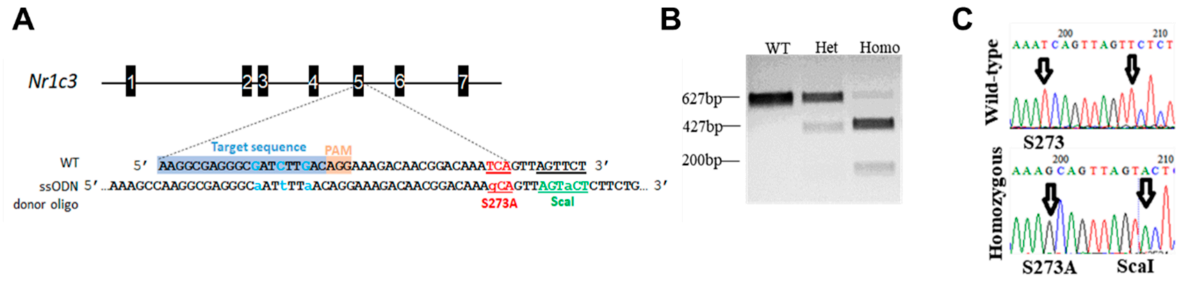

Table S1. Composition of diets. Composition of high-fat high-sucrose, maintenance and high-fat diets, with percentage of carbohydrates, proteins and fat, and total of kcal for each diet.

	High-fat High-sucrose diet (HFHS)	Maintenance diet (MNT)	High-fat diet (HFD)
**% Carbohydrates/kg diet**	44	64	20
**% Proteins/kg diet**	14	14.1	20
**% Fat/kg diet**	42	10	60
**Total Kcal/kg diet**	4762.5	3601.0	5217.3

Figure S2. Experimental design of the study. The experimental design involved the use of different diets and time periods to evaluate the effects of short and prolonged high-fat feeding. Male WT and KI mice were weighed, and their glycemia was measured at weaning (3 weeks old). Blood was collected from the tail to obtain plasma, and insulin levels were measured. After weaning, the animals were divided into two groups. Group 1 was fed with MNT and HFHS diets for 8 weeks and then euthanized at 11 weeks old.Group 2 was fed with MNT and HFD diets for 18 weeks, starting with MNT diet from weaninguntil 6 weeks old and then HFD diet until euthanasia at 24 weeks old. In both groups, we performed insulin and glucose tolerance tests (ITT and GTT, respectively) and collected liver, white and brown adipose tissues (WAT and BAT, respectively), gastrocnemius muscle, kidney, heart (only for HFD feeding), and blood for plasma analysis on the euthanasia day. The animals were housed in groups of 3-5 per cage and maintained on a photoperiod of 12:12light/dark cycle, at 21-24°C, with free access to food and water.

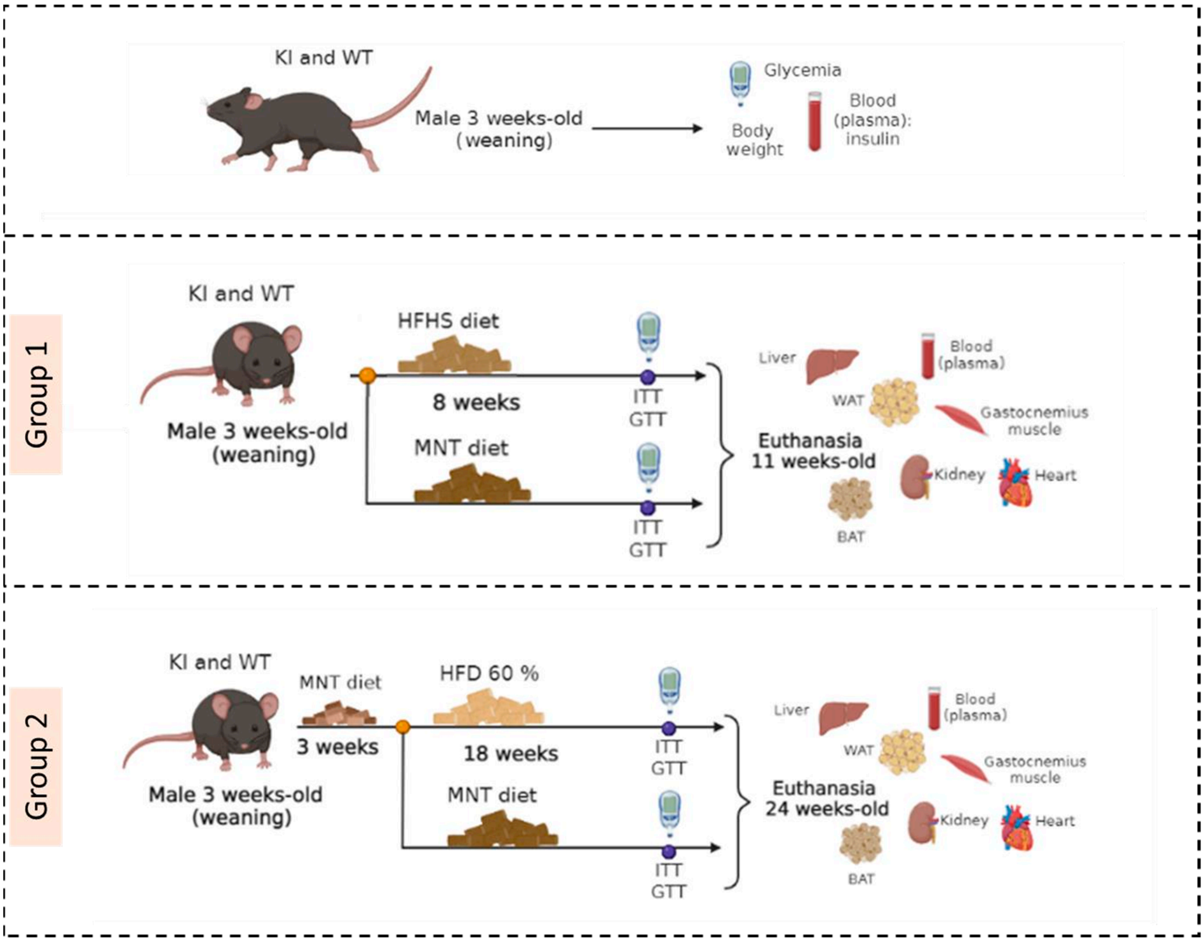

Table S2. Sequence of primers and their standardizations. Primers used forgene expression on qPCR, with each forward and reverse sequences, reference numbers from gene bank, optimized concentration, percentage of efficiency and r².

Gene	Primer sequence<br>Forward<br>Reverse	Reference from gene bank	Optimized concentration	% eficiency	R²
Rpl27	CTGGCCTTGCGCTTCAA<br>TCATGCCCACAAGGTACTCTGT	NM_011289.3	500nM	96.3	0.99
36b4	GAGGAATCAGATGAGGATATGGGA<br>AAGCAGGCTGACTTGGTTGC	NM_007475.5	300nM	99.6	0.99
Txnip	TATGTACGCCCCTGAGTTCC<br>GCTCACTGCACGTTGTTGTT	NM_001009935.2	300nM	104.99	0.99
Nr1d2	ATGTCACGAGATGCTGTTCG<br>TGGTCTTCATTGCACTTTGC	NM_011584.4	500nM	104.9	0.99
Lep **(Leptin)**	GAGACCCCTGTGTCGGTTC<br>CTGCGTGTGTGAAATGTCATTG	NM_008493.3	500nM	98.3	0.98
Cfd **(Adipsin)**	AATCTGCGCACGTACCATGA<br>AACCACACCTTCGACTGCAT	NM_001291915.2	500nM	95.4	0.99
Gdf3	AACTTCTGCCACCGTCATCA<br>ATCAGAGCCTGCATGAAAGC	NM_008108.5	300nM	100	0.99
Ppm1a	TGAAATGGAGGACGCACACA<br>CTCACAGCAGTATTTGGCAACC	NM_008910.3	600nM	100	0.99
Npy	TCGTGTGTTTGGGCATTCTG<br>TGAAATCAGTGTCTCAGGGCTG	NM_023456.3	500nM	97.9	0.99
Ucp1	TGTTCATTGGGCAGCCTACA<br>ACAAGCTTTCTGTGGTGGCT	NM_009463	300nM	98.5	0.98
Crh	AGATGTACAGGGAGAGAGCCTA<br>TCCTTGGGGCCACATTTTCT	NM_205769.3	500nM	99.6	0.97
Pdk4	AGCCTATGTGCAAACCCAGA<br>AACATTTCACCCAAGCCTCG	NM_013743.2	300nM	97.4	0.99

Fabp4	AAAGAAGTGGGAGTGGGCTT<br>CACGCCCAGTTTGAAGGAAA	NM_024406.3	300nM	99.5	0.99
CD36	GCTGTGTTTGGAGGCATTCT<br>TGCCACGTCATCTGGGTTTT	NM_001159555.1	600nM	97.3	0.99
TNFα	CCCTCACACTCAGATCATCTTCT<br>GCTACGACGTGGGCTACAG	NM_013693.3	300nM	98.4	0.99
PPARγ	AGGGCGATCTTGACAGGAAA<br>TCGAAACTGGCACCCTTGAA	NM_001127330.2	500nM	99.2	0.99

Figure S3. Data of WT and KI animals after 8 weeks of maintenance (MNT) diet. KI S273A lineage after 8 weeks of maintenance diet (MNT), since weaning, comparing with WT animals. (A) Body weight gain during the 8 weeks of MNT diet. (B) Intraperitoneal insulin tolerance test (ipITT) with its respective area under the curve (AUC). (C) Intraperitoneal glucose tolerance test (ipGTT) with its respective AUC. (D) Plasma cholesterol and (E) triglycerides levels on fasted state. (F) Plasma cholesterol levels on fed state. (G) Hepatic cholesterol and (H) triglycerides. Data are represented as mean ± SEM. Statistical analysis was done using non-parametric t test. $n \geq 5$ per group.

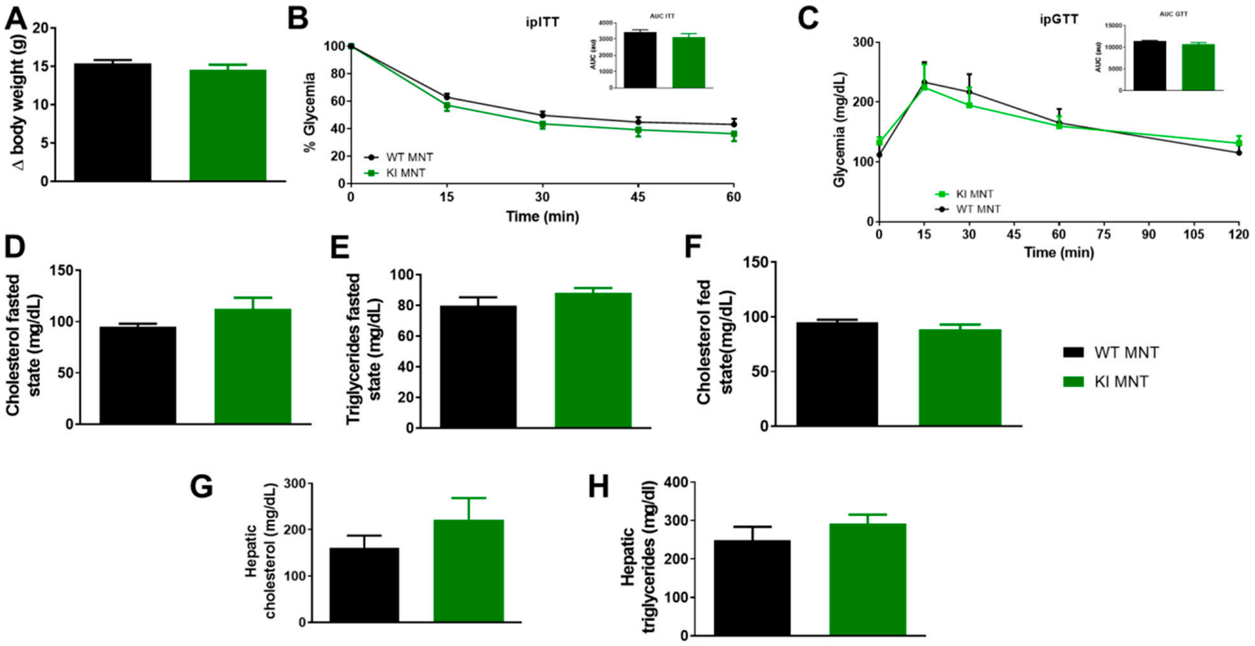

Figure S4. Data of WT and KI animals after 8 weeks of high-fat high-sucrose(HFHS) diet. KI S273A lineage after 8 weeks of high-fat high-sucrose diet (HFHS), since weaning, comparing with WT animals. (A) Food intake and (B) body weight gain during the 8 weeks of HFHS diet. (C) Glycemia and (D) insulin levels on fasted state. (E) Glycemia and
(F) insulin levels on fed state. (G) Plasma cholesterol and (H) triglycerides levels on fasted state. (I) Plasma cholesterol levels on fed state. (J) Hepatic cholesterol and (K) triglycerides. Data are represented as mean ± SEM. Statistical analysis was done using non-parametric t test. *$p<0.05$, **$p<0.01$. $n \geq 5$ per group.

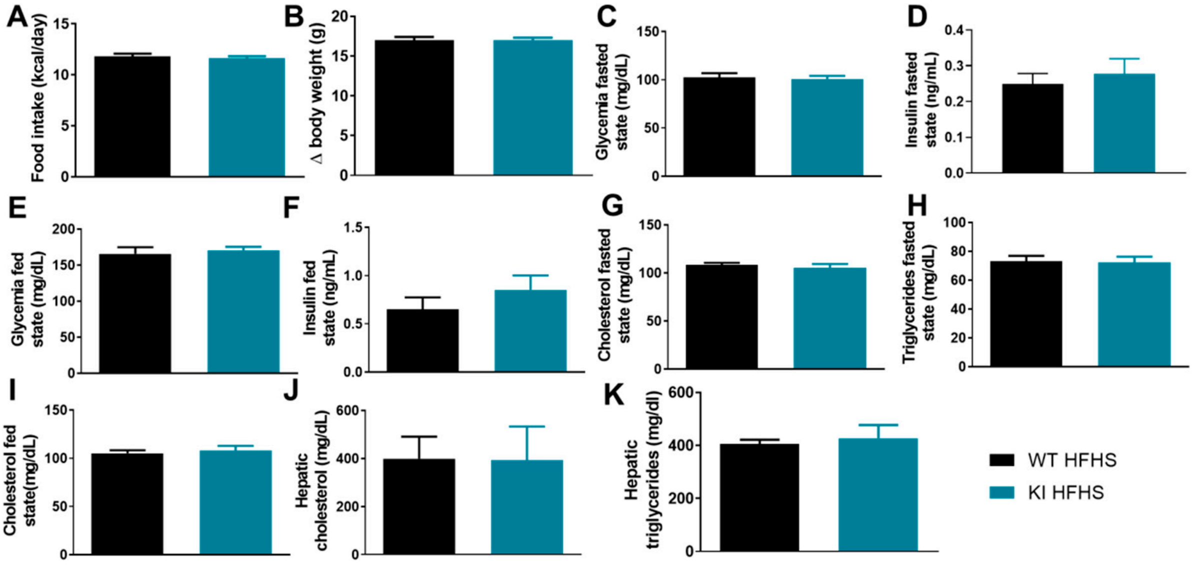

Figure S5. Data of WT and KI animals after 18 weeks of high-fat diet (HFD). Characterization of KI S273A lineage during 18 weeks of high-fat diet (HFD) and after two weeks of vehicle treatment. (A) Evolution of absolute body weight from weaning until 22 weeks-old, with 16 weeks of HFD beginning at 6 weeks-old. (B) Percentage of weight gain and (C) food intake for 18 weeks of HFD. (D) Fed glycemia obtained from tail vein on euthanasia day using glycosometer after 18 weeks of HFD. (E) Organs weight on euthanasia day: epididymal white adipose tissue (eWAT), liver, heart, kidney, brown adipose tissue (BAT) and gastrocnemius. Data are represented as mean ± SEM. Statistical analysis was done using non-parametric t test. *p<0.05, **p<0.01, ***p<0.001 and ****p<0.0001. *n*≥5 per group.

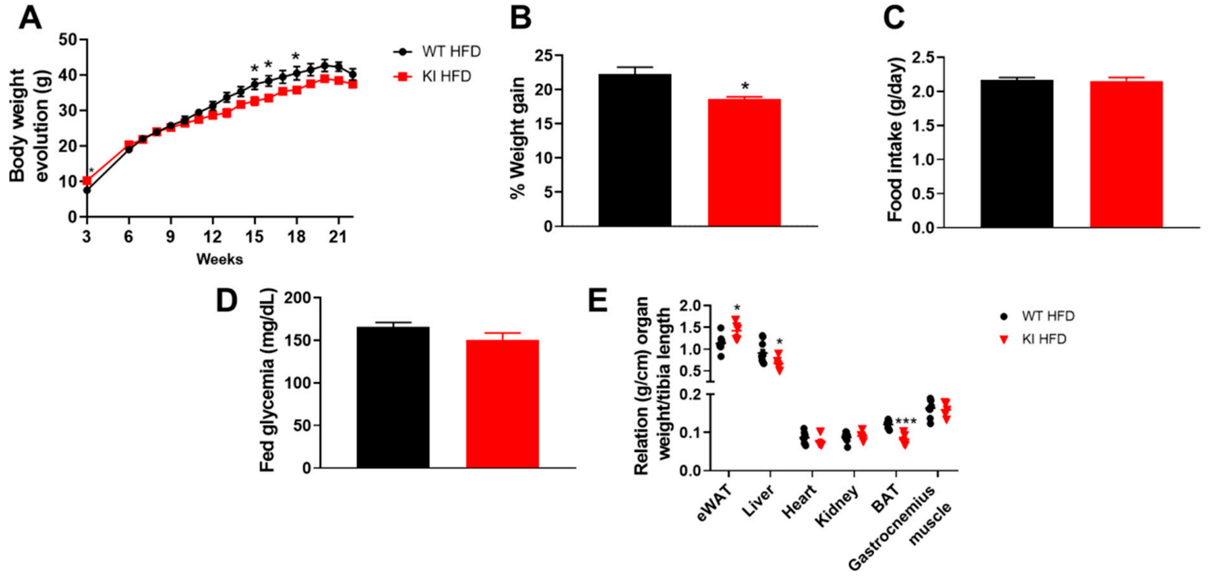

Table S3. Adipocytes analysis. Data of area and perimeter of adipocytes on each group: number of cells (n), average, first and third quartile, and this last one was used as reference for removing outliers (2*IQR) Statistical analysis was done using Bayesian non- parametric test. *n*≥5 per group.

**Variable**	**Group**	**n**	**Average**	**Q1**	**Q3**	**IQR**	**BF10 (*vs.* vehicle)**
**Area (μm$^{2)}$**	Vehicle	85495	3603.6	965.9	5460.9	4495	-
	Knockin	20997	4201.3	1823.1	5921.3	4098.3	301.89
**Perimeter (μm)**	Vehicle	85495	278.9	168.6	367.9	199.4	-
	Knockin	20997	339.1	225.1	433.6	208.5	∞

Figure S6. Original membranes of western blotting analysis of Ser273 phosphorylation and PPARγ. Western Blotting membranes for PPARγ, pSer273 and vinculin. Total protein extracted from eWAT, *n*≥3 per group.

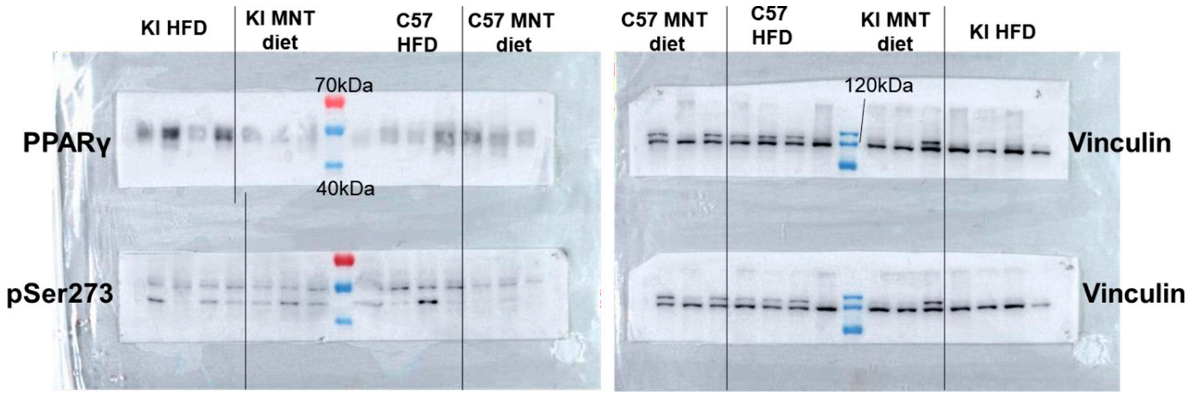

Supplement: Supplementary file 1 [file biomolecules-13-00632-s001.zip › biomolecules-2194659-supplementary.pdf]
